# PpCBF3 from Cold-Tolerant Kentucky Bluegrass Involved in Freezing Tolerance Associated with Up-Regulation of Cold-Related Genes in Transgenic *Arabidopsis thaliana*

**DOI:** 10.1371/journal.pone.0132928

**Published:** 2015-07-15

**Authors:** Lili Zhuang, Xiuyun Yuan, Yu Chen, Bin Xu, Zhimin Yang, Bingru Huang

**Affiliations:** 1 College of Agro-grassland Science, Nanjing Agricultural University, Nanjing, Jiangsu, PR China; 2 Department of Plant Biology and Pathology, Rutgers University, New Brunswick, NJ 08901, United States of America; Chinese Academy of Sciences, CHINA

## Abstract

Dehydration-Responsive Element Binding proteins (DREB)/C-repeat (CRT) Binding Factors (CBF) have been identified as transcriptional activators during plant responses to cold stress. The objective of this study was to determine the physiological roles of a CBF gene isolated from a cold-tolerant perennial grass species, Kentucky bluegrass (*Poa pratensis L*.), which designated as *PpCBF3*, in regulating plant tolerance to freezing stress. Transient transformation of *Arabidopsis thaliana* mesophyll protoplast with PpCBF3-eGFP fused protein showed that PpCBF3 was localized to the nucleus. RT-PCR analysis showed that *PpCBF3* was specifically induced by cold stress (4°C) but not by drought stress [induced by 20% polyethylene glycol 6000 solution (PEG-6000)] or salt stress (150 mM NaCl). Transgenic Arabidopsis overexpressing *PpCBF3* showed significant improvement in freezing (-20°C) tolerance demonstrated by a lower percentage of chlorotic leaves, lower cellular electrolyte leakage (EL) and H_2_O_2_ and O_2_^.-^ content, and higher chlorophyll content and photochemical efficiency compared to the wild type. Relative mRNA expression level analysis by qRT-PCR indicated that the improved freezing tolerance of transgenic Arabidopsis plants overexpressing *PpCBF3 *was conferred by sustained activation of downstream cold responsive (COR) genes. Other interesting phenotypic changes in the *PpCBF3-*transgenic Arabidopsis plants included late flowering and slow growth or ‘dwarfism’, both of which are desirable phenotypic traits for perennial turfgrasses. Therefore, *PpCBF3* has potential to be used in genetic engineering for improvement of turfgrass freezing tolerance and other desirable traits.

## Introduction

Low-temperature stresses such as chilling (0–20°C) and freezing stress (< 0°C) are the most common environmental factors limiting plant growth and productivity in cool climatic regions [1,2]. During chilling stress and the thawing phase following freezing stress, cells loss water due to the rupture of cellular membranes, leading to dehydration and therefore, protecting cellular dehydration is critically important for plant survival of chilling or freezing stress [2]. Plant adaptation to chilling or freezing stress involves various molecular changes, including the induction of many low-temperature response genes and transcription factors, such as dehydration-responsive-element-binding protein (DREB1)/C-repeat binding factors (CBFs) [3,4]. DREB1/CBFs are known to function in regulating plant responses to low temperatures which may induce cellular dehydration [5,6].

DREB1/CBFs are a family of genes conserved in flowering plants and CBF homolog genes have been identified and analyzed in many agronomic crops, such as maize (*Zea mays*), rice (*Oryza sativa*), wheat (*Triticum aestivum*) and soybean (*Glycine max*) [7–10]. DREB1/CBFs regulate the downstream expression of cold-inducible genes, including cold-regulated genes (COR) [4,11,12]. Enhanced cold tolerance was achieved with DREB1/CBF genes constitutively expressed at high levels in agriculturally important species (i.e. rice and wheat) and model species [i.e. Arabidopsis and tobacco (*Nicotiana benthamiana*)] [13–18]. For example, transgenic rice plants overexpressing OsDREB1 showed higher survival rate to chilling (2°C) stress compared to the wild type [13]. Transgenic barley (*Hordeum vulgare*) plants overexpressing *TaDREB2* and *TaDREB3* showed increased survival rate under frost stress (-6°C) [15]. Heterologenic expression of a CBF3 gene isolated from sweet pepper (*Capsicum annuum*) conferred enhanced tolerance to chilling stress (4°C) in transgenic tobacco plants through higher accumulations of osmolytes (proline and soluble sugars), higher levels of unsaturated fatty acids, and lower accumulations of H_2_O_2_ and O^2.-^, as well as lower electrolyte leakage and photochemical efficiency [18]. Overexpression of CBF1 enhanced Arabidopsis tolerance to freezing stress (-8°C) as by lower electrolyte leakage and improved whole plant survival [19]. Similarly, overexpressing CBF3 lead to enhanced tolerance to freezing (-6°C) stress by activating downstream cold responsive genes [20]. However, constitutive overexpression of *LeCBF1* or *AtCBF3* genes had no effects for improving freezing tolerance in freezing-sensitive tomato (*Lycopersicon esculentum*) due to different CBF regulon [21].In general, it is obvious that the extent of DREB1/CBF affecting plant tolerance to cold stress varies with the level of temperature stress, specific type or group of the gene family, and plant species where the genes are isolated from [8,18,21–23]. Furthermore, most of previous studies examined plant responses to chilling stress or sub-freezing temperatures. Increasing frequency of extremely low temperature below 0°C occurs in cool-climatic regions associated with global climate changes [1]. Nevertheless, the knowledge of DREB1/CBFs transcriptional control of plant tolerance to severe freezing temperatures is limited.

Cool-season perennial grasses used as forage or managed turf are typically exposed to sub-zero freezing temperatures during winter months though limited information is available as to how changes in DREB1/CBF genes may confer cold tolerance and improved survivability. DREB1/CBF genes were identified in perennial ryegrass (*Lolium perenne*) and tall fescue (*Festuca arundinacea*) [23–25]. *LpCBF3* was induced by chilling stress (4°C) and transgenic Arabidopsis plants overexpressing *LpCBF3* showed higher survival rate at -6°C [23,25]. However, physiological roles and molecular mechanisms of DREB1/CBF genes isolated from cold-tolerant perennial grasses have not been well documented and is a topic which deserves further investigation.

The DREB1/CBF gene (*PpCBF3*) was isolated from Kentucky bluegrass (*P*. *pratensis*), a species which displays exceptional cold tolerance [26], to investigate whether *PpCBF3* could positively regulate plant tolerance to severe freezing stress (-20°C). The objectives of this study were to determine the specificity of *PpCBF3* from Kentucky bluegrass for cold stress responses by characterizing the expression patterns of *PpCBF3* in response to different abiotic stresses and to examine physiological functions and downstream genes regulated by *PpCBF3* conferring plant tolerance to freezing stress (-20°C) through overexpressing it in Arabidopsis.

## Materials and Methods

### Growth conditions for Kentucky bluegrass seedlings

Seeds of Kentucky bluegrass cultivar (*Poa pratensis* ‘Midnight’) were germinated on Petri dishes in darkness in a growth chamber (MT8070iE, Xubang, Henan, China) set to day/night temperature of 25/15°C and humidity of 70%. 7 d old seedlings were transferred to plastic pots filled with a mixture of moss peat (Pindstrup Sphagnum, Ryomgaard, Denmark) and vermiculite (3:1 by volume) and maintained in a greenhouse with natural sunlight and an average day/night temperature of 23/18°C. Plants were irrigated every other day and fertilized weekly with half-strength Hoagland’s nutrient solution [27], and trimmed regularly to keep the plant height at about 5 cm.

### Isolation *PpCBF3* gene from Kentucky bluegrass

For gene isolation, 4-week-old Kentucky bluegrass seedlings were subjected to 4°C in an incubator with cool-white fluorescent lights for 1 h. Total RNA was isolated from leaves using Rnapure fast isolation Kit (YuanPingHao, Tianjin, China) and cDNA was synthesized by Transcriptor First Strand cDNA Synthesis Kit (Roche, Rotkreuz, Switzerland). RACE (rapid amplification of cDNA ends) PCR was carried out to extend the 3’ region of the *PpCBF3* by using gene specific primer 1 (GSP1F1) (S1 Table) for the first cycle and GSP2F2 for the second cycle with each paired to an adaptor-specific primer (Adaptor-R). Degenerate primer from start codon ATG was designed according to *CBF3* sequence from other monocot plants and the 5’-end sequence of *PpCBF3* was obtained by using forward primer degF and reverse primer degR. Full-length CDS of *PpCBF3* was amplified using conCBF3F and conCBF3R primers. The PCR amplification procedure was first initial denaturation at 94°C for 5 min, with 35 cycles (94°C for 30 s, 60°C for 30 s, 72°C for 1 min) followed, and a final extension cycle of 72°C for 7 min. PCR fragment obtained was cloned into pMD19-T Vector (Takara, Kyoto, Japan). Positive clones confirmed by colony PCR were sequenced (Springen Co., Ltd, Nanjing, China).

### RT-PCR and qRT-PCR analysis of *PpCBF3* gene expression in response to abiotic stresses

In order to determine whether *PpCBF3* is specifically responsive to cold stress or broadly responsive to different abiotic stress, *PpCBF3* gene expression patterns were analyzed for plants exposed to cold, drought and salt stress. For drought and salt treatment, tillers of Kentucky bluegrass were transferred into half-strength Hoagland’s nutrient solution containing PEG-6000 (20% w/v) to induce drought stress or NaCl (150 mM) to induce salt stress under normal growth temperature (a day/night temperature of 25/15°C). For cold treatment, Kentucky bluegrass plants grown in plastic pots were treatted in an incubator at 4°C. To examine gene responses to different stresses, leaf samples were collected at 0, 15 min, 30 min, 1 h, 3 h, 6 h, 12 h and 24 h of stress treatment and frozen in liquid nitrogen.

For qRT-PCR analysis, total RNA was extracted using a Tripure Isolation Reagent Kit (Roche Diagnostic, Basel, Switzerland) according to the manufacturer’s protocol. The first strand cDNA was synthesized with 1 μg RNA using the PrimeScript RT reagent Kit with gDNA Eraser (Perfect Real Time) (Takara, Otsu, Japan). qRT-PCR was performed on Roche LightCycler480 II machine (Roche Diagnostic, Rotkreuz, Switzerland) with SYBR Green I Master reaction system (Roche Diagnostic, Rotkreuz, Switzerland). The PCR condition was as follows: initial denaturation step of 10 min at 95°C, 40 cycles of PCR (95°C for 15 s, 60°C for 15 s, and 72°C for 20 s). Data was collected at 65°C in each cycle. The qRT-PCR analysis used three biological replicates for every treatment, each with three technical replicates. Relative expression level of genes was determined by 2^-ΔΔCT^ method using *P*. pratensis elongation factor 1α (*PpEF1α*) as the reference gene.

For RT-PCR analysis, total RNA extraction and first strand cDNA synthesis was done as previously described and primers for reference gene *PpEF1α* were the same in qRT-PCR.

### Isolation and transient transformation of Arabidopsis mesophyll protoplast

For gene transformation and a phenotypic control, *Arabidopsis thaliana* accession Columbia (col-0) was used. Seeds of Arabidopsis were treated with 1.3% NaClO for 15 min and washed thoroughly with sterile water six times. Seeds were sowed on MS medium [28] with 0.2% low melting-point agarose for uniform separation, vernalized in darkness for 72 h at 4°C, and grown on MS medium at 23°C with a 16 h photoperiod under 120 μmol m^-2^ s^-1^ light density (designated as normal condition) in a controlled growth chamber (Haier, Qingdao, China).

Arabidopsis protoplasts were isolated according to Wu et al. (2009) [29]. The upper and lower epidermal surface of arabidopsis leaves was affixed to Time tape (Time Med, Burr Ridge, IL) and Magic tape (3 M, St. Paul, MN), respectively. The lower side of epidermal cell layer was peeled by pulling off the Magic tape. The peeled leaves were immersed in 10 ml enzyme solution (with lower surface in the sulution) [1% cellulase ‘Onozuka’ R10 (Yakult, Tokyo, Japan), 0.25% macerozyme ‘Onozuka’ R10 (Yakult), 0.4 M mannitol, 10 mM CaCl_2_, 20mM KCl, 0.1% BSA and 20 mM MES, PH 5.7] for 1 h in light, the protoplasts released into solution were collected by centrifuged at 100 ×g for 3 min and washed twice with prechilled W5 solution [154 mM NaCl, 125 mM CaCl_2_, 5 mM KCl, 5 mM glucose, and 2mM MES, PH 5.7] and incubated on ice for 30 min. Protoplasts were finally resuspended in modified MMg solution (0.4 M mannitol, 15 mM MgCl_2_, and 4 mM MES, PH 5.7) with a concentration of 5 ×10^5^ cells mL^-1^.

To investigate the subcellular localization of PpCBF3 in living cells, the ORF (open reading frame) of *PpCBF3* without a stop codon in a pENTR1A Dual Selection Vector (Invitrogen, Carlsbad, CA, USA) was LR-recombinated with a small binary vector P2GWF7.0 (~6.7 Kb). Thus PpCBF3 was fused to the N-terminal of eGFP reporter gene under the cauliflower mosaic virus 35S (CaMV35S) promoter. About 30 μg *PpCBF3-eGFP* recombinant plasmids were transiently transformed into Arabidopsis protoplasts by the PEG4000 (Fluka, USA)-mediated method [29]. After incubating in 6-well plates at 25°C for 16 h in light, photographs were captured by Confocal Laser Scanning Microscope (Carl Zeiss, Jena, Germany).

### Construction of plant expression vector and transformation of *Arabidopsis thaliana*

The ORF of *PpCBF3* with *Eco*RI and *Eco*RV sites was first cloned into a pENTR1A Dual Selection Vector and transformed into pEarleyGate 103 destination plasmid using LR Clonase II enzyme mix (Invitrogen, Carlsbad, CA, USA) [30] with CaMV35S as promoter. The *CaMV35S*::*PpCBF3* constructs were introduced into *Agrobacterium tumefaciens* strain EHA105. Floral dip method [31] was adopted to obtain transgenic Arabidopsis. Seeds (T_1_) from transgenic plants were selected on MS medium [with 20 μg ml^-1^ glufosinate ammonium (Sigma, USA) supplemented]. The glufosinate ammonium-resistant T_1_ seedlings were tested by PCR analysis using the primers for *PpCBF3* (conCBF3R) and pEarleyGate103 expression vector (103F). T_2_ seeds produced from the T_1_ plants expressing *PpCBF3* gene were collected for further analysis.

### Phenotypic analysis of transgenic Arabidopsis under normal temperature and freezing stress

T_2_ seeds of transgenic lines were germinated on MS medium (supplemented with 20 μg ml^-1^ glufosinate ammonium) to select for positive transgenic plants and WT seeds were germinated on MS medium concurrently. One-week-old seedlings of transgenic plants and WT were then transplanted to new MS medium in Petri dishes and grown at normal condition (23°C with a 16 h photoperiod under 120 μmol m^-2^ s^-1^ light densities). Freezing tests were performed following the protocol by Li et al. (2014) [32] with some modifications. 4-week-old transgenic lines and WT plants were acclimated at 4°C in an incubator with cool-white fluorescent lights for 3 d, and subsequently exposed to freezing temperatures gradually decreasing from -2 to -20°C within 2 h and finally at -20°C for 3 h in a freeze chamber (Beckman Coulter, Brea, CA). Plants were then moved to 4°C for 24 h and recovered at 23°C for 7 d.

The typical symptoms of freezing injury include loss of chlorophyll and membrane stability [33]. Therefore, several commonly used stress indicators, the percentage of chlorotic leaves per plant, leaf chlorophyll content (Chl), leaf photochemical efficiency, and membrane stability, were measured after plants were thawed following the freezing treatment to evaluate whether over-expressing *PpCBF3* would alleviate freezing damages.

The percentage of leaf chlorosis was calculated based on the number of leaves displaying yellow divided by the total number of leaves per plant. Photographs of plants were taken using single lens reflex camera (Nikon D5100, Thailand).

For phenotype analysis under normal condition, 14-day-old Arabidopsis seedlings were transplanted into plastic pots and maintained at normal growth condition for up to 6 weeks. The number of rosette leaves per plants was counted and photographs of plants were taken using the camera described above.

Leaf Chl was calculated as described in Arnon (1949) [34]. First fully-expanded leaves (0.1 g) from plant top were detached from plants and soaked in 95% dimethylsulfoxide in darkness for about 72 h in order to extract chlorophyll completely. The absorbance of Chl extract was measured at 663 and 645 nm using a spectrophotometer (GE Healthcare Life Sciences, Cambridge, UK).

Leaf photochemical efficiency expressed as the ratio of variable to maximum fluorescence (Fv/Fm) was determined in both transgenic lines and WT exposed to freezing stress. Leaf Fv/Fm was measured with a fluorescence induction monitor (OPTI-Sciences, Hudson, USA) following 30 min dark adaptation.

Cellular membrane stability was measured as leaf electrolyte leakage (EL). Fully-expanded leaves (about 0.1 g) of WT and transgenic lines were detached and incubated in 15 ml distilled deionized water. The initial level of EL (Ci) was measured using a conductance meter (Thermo Scientific, Baverly, USA) after shaken for 24 h at room temperature. Leaf tissue was killed in an autoclave at 121°C for 30 min. The conductance of the incubation solution was measured after 24 h incubation on a shaker. Relative EL was calculated as EL = (Ci/Cmax) × 100 [35].

### Histochemical detection of H_2_O_2_ and O_2_^.-^ in transgenic plants exposed to freezing stress

The production of hydrogen peroxide (H_2_O_2_) in leaves exposed to -20°C for 1 h (firstly acclimated at 4°C for 24 h) and thawed at 4°C for 24 h was detected by 3, 3’-diaminobenzidine (DAB) staining as described by Lee et al. (2002) [36]. Three-week-old seedlings of transgenic and WT lines were vacuum-incubated with 0.1 mg ml^-1^ DAB (sigma, USA) in 50 mM Tris-acetate buffer (pH 5.0). Solutions with seedlings were incubated at 25°C in the darkness for 24 h. Chlorophyll was removed by incubation in 70% ethanol at room temperature for 24 h.

Detection of superoxide free radicals were performed as described previously [36]. All seedlings acclimated at 4°C for 24 h, then exposed to freezing stress (-20°C for 1 h) and thawed at 4°C for 24 h were harvested, and vacuum-infiltrated [0.1 mg ml^-1^ nitroblue tetrozolium (NBT) in 25 mM HEPES buffer (pH7.6)]. Samples were subsequently incubated at 25°C for 2 h in the darkness. Stained samples were bleached in 70% ethanol and incubated at 25°C for 24 h to thoroughly remove the chlorophyll. Photographs were taken using a stereomicroscope (Olympus, Tokyo, Japan).

### qRT-PCR analysis of *PpCBF3* gene and downstream genes in transgenic plants and WT in response to cold stress

Leaves of 3-week-old transgenic and WT plants exposed to 4°C in a freezer with cool-white fluorescent lights for 4 h were harvested and frozen in liquid nitrogen for further analysis. For qRT-PCR analysis in Arabidopsis, methods were the same as mentioned above (S1 Table). 2^-ΔΔCT^ method was adopted to calculate the expression levels of genes with *AtActin2* as the reference gene. The qRT-PCR analysis had three biological replicates for every treatment, each with three technical replicates.

### Western blot analysis of CBF protein in PpCBF3-transgenic and WT Arabidopsis

Crude proteins of transgenic and WT lines were extracted with phosphate buffer (pH 7.0), incubated on ice for 0.5 h and centrifuged at 12,000 g for 30 min. Protein samples were separated by 12% SDS-PAGE. The proteins were semi-dry blotted (Amersham Biosciences, Piscataway, USA) onto a PVDF membrane (Roche, Basel, Switzerland). For detection, the membranes were incubated for 2 h with the Anti-GFP mouse monoclonal antibody (1:5000) (CMCTAG, Milwaukee, USA) after incubation overnight at 4°C in blocking buffer (5% non-fat dried milk in PBST). Membranes were then rinsed 4 times in PBST and incubated with the secondary antibody [horseradish-peroxidase conjugated goat anti-mouse antibody (Abgent, San Diego, America) 1: 5 000 in blocking buffer] for 1 h. Finally, the membranes were incubated with Pierce ECL Western Blotting Substrate (Thermo Scientific, Rockford, USA) and detected by a CCD Video Camera Imaging System (Vilber Lourmat, Marne la Vallée, France). The contrast and brightness of resulting pictures were processed with BIO-1D software (Vilber Lourmat, Marne la Vallée, France).

### Statistical analysis of phenotypic and gene expression data

Physiological data and qRT-PCR data were subjected to the analysis of variance (ANOVA) analysis of variance according to the general linear model procedure of SAS (SAS 9.0, Cary, NC). Differences between the means were distinguished by Fisher’s protected least significance difference (LSD) test at the 0.05 probability level.

## Results

### Isolation of *PpCBF3* from Kentucky bluegrass and subcellular localization of PpCBF3-eGFP fused protein

**Fig 1:**
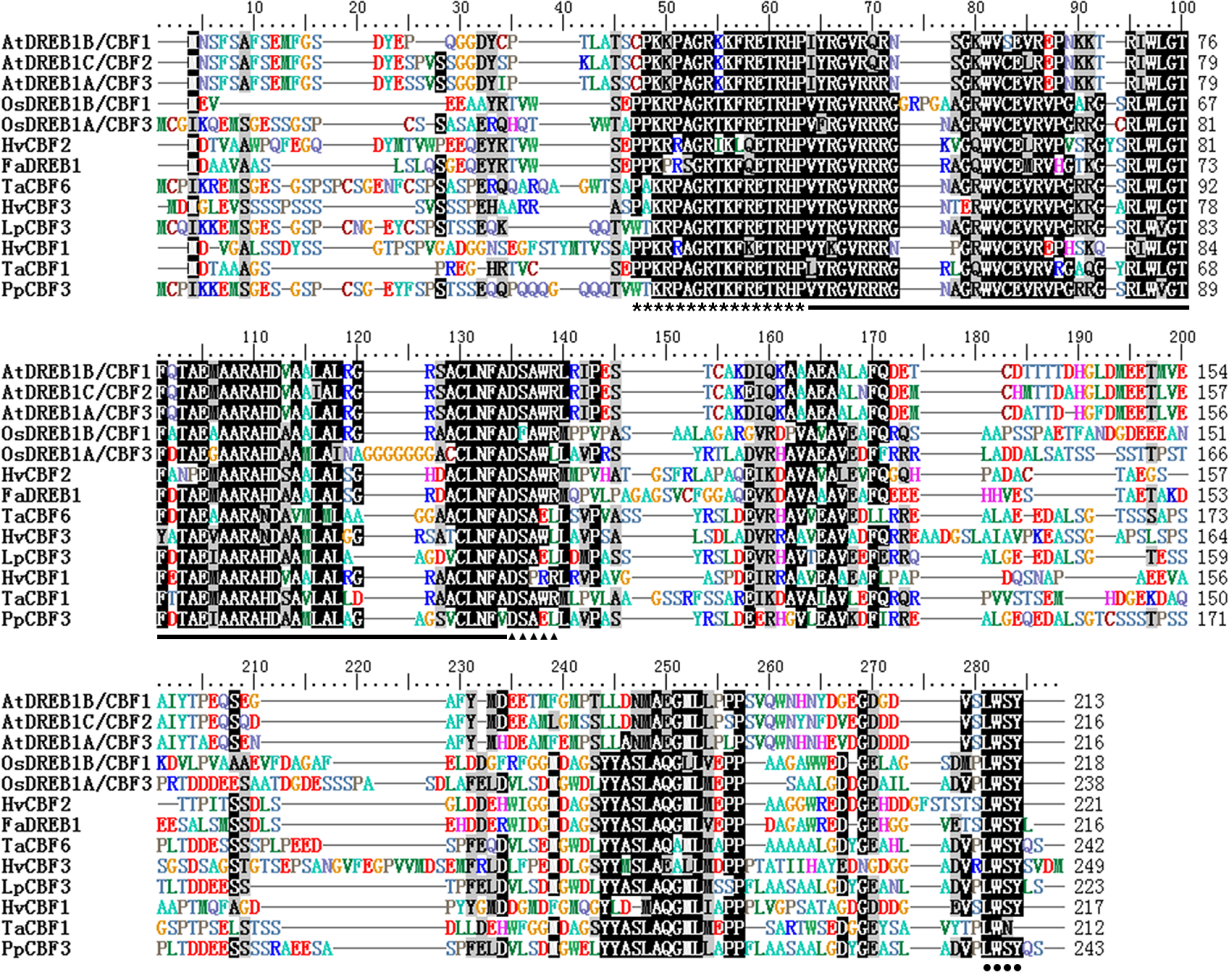
Sequence homology analysis of PpCBF3 with DREB/CBFs from Arabidopsis, rice, wheat, barley, tall fescue and perennial ryegrass. Conserved AP2 DNA-binding domain is indicated by black line, two signature sequence of CBF are marked by asterisk and filled triangle. ‘LWSY’ domain at the end of C terminal is indicated by black dots. Accession numbers of the proteins are as follows: AtDREB1B/CBF1 (AAC49662), AtDREB1C/CBF2 (AAD15976), AtDREB1A/CBF3 (AAD15977), OsDREB1B/CBF1 (AAN02488), OsDREB1A/CBF3 (AAN02486), HvCBF2 (AAM13419), FaDREB1 (AAQ98965), TaCBF6 (AAX28964), HvCBF3 (AAG59618), LpCBF3 (AAX57275), HvCBF1 (AAL84170), TaCBF1 (AAL37944), PpCBF3 (KP258182). Sequence alignment was done by Cluster W.

**Fig 2:**
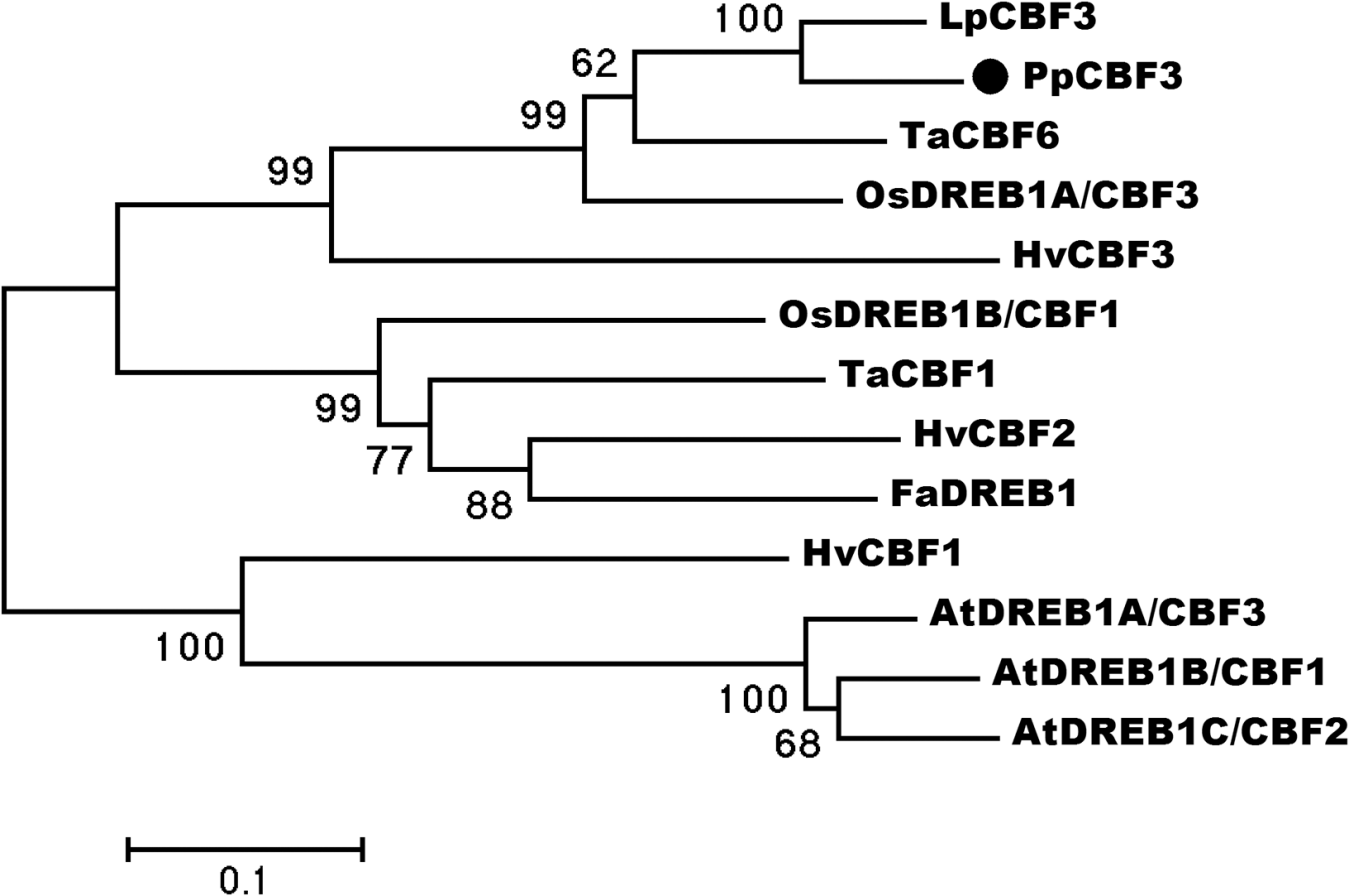
Phylogenetic tree of PpCBF3 and other DREB/CBF proteins. The tree is constructed by MEGA 4.0 software (Tamura et al. 2007) based on alignment of complete protein sequences. Black dot indicates PpCBF3 protein. Accession number of protein sequences used here are the same as in Fig 1.

A 569 bp nucleotide fragment obtained by 3’ RACE and 5’-end of cDNA was extended by homology-based cloning. Finally, a full-length sequence consisted of 860 bp nucleotides with a 732 bp ORF encoding a 243-residue polypeptide was obtained. Sequence analysis at http://blast.ncbi.nlm.nih.gov/Blast.cgi by Blastx programme showed that the gene had high similarity to DREB1/CBF in other plant species (Fig 1); about 81% similarity to *LpCBF3* in perennial ryegrass and 64% to *OsDREB1A/CBF3* in rice. A phylogenetic tree revealed that the gene is a putative ortholog of *LpCBF3* (Fig 2). We designated the gene as *PpCBF3* and the accession number in NCBI database was KP258182. In addition to an AP2 conserved domain serving a DNA-binding role, two signature sequences (PKK/RPAGRxKFxETRHP and DSAEL) also presented in PpCBF3. The C-terminal LWSY motif was also conserved in PpCBF3 compared with other CBF-like proteins (Fig 1). The *PpCBF3* isolated from Kentucky bluegrass encoded an ortholog of *CBF3*-like transcription factor.

**Fig 3:**
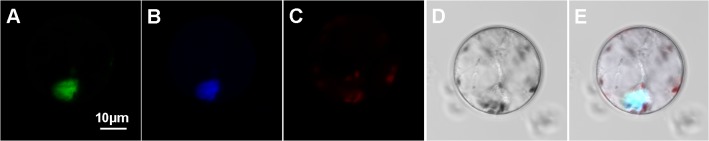
Subcellular localization of PpCBF3 protein. (A) Fluorescence image of Arabidopsis mesophyll protoplast expressing the PpCBF3-eGFP fusion protein. (B) Fluorescence image of nucleus in protoplast stained with DAPI. (C) Fluorescence image of mesophyll in Arabidopsis protoplast. (D) Image of Arabidopsis mesophyll protoplast under bright field. (E) Merged fluorescence image of Arabidopsis protoplast expressing the PpCBF3-eGFP fusion protein and stained with DAPI. (A)-(C), dark field. (D), (E), bright field. All bar = 10 μm.

As shown in Fig 3, the GFP signal was confined predominantly to the nucleus and was merged with DAPI in the individual cells transferred with the *PpCBF3-eGFP* recombinant plasmids. PpCBF3 was present as a nuclear protein.

### *PpCBF3* expression in response to abiotic stresses

**Fig 4:**
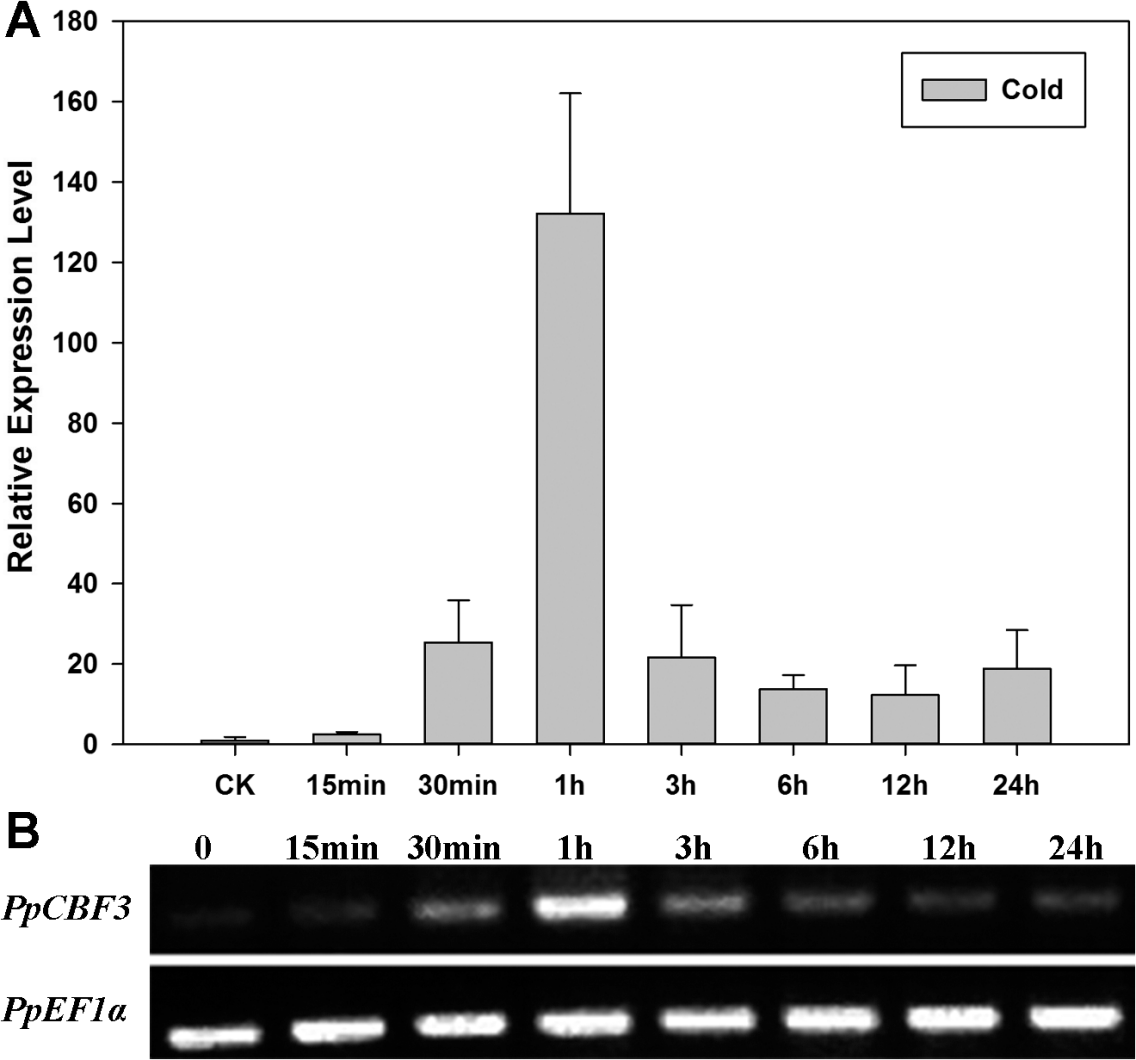
Relative mRNA expression level of *PpCBF3* in leaves of *P*. ***pratensis* at different time scale under 4** ^**o**^ **C by RT-PCR and qRT-PCR.** (A) qRT-PCR analysis of *PpCBF3* expression level. Three independent experiments show the similar results and here shows one of the results. Values are means ± SD of three technical repetitions. (B) RT-PCR analysis of *PpCBF3* expression level.

qRT-PCR analysis demonstrated that *PpCBF3* exhibited a rapid response to cold stress (4°C), as the expression level increased within 15 min, reached to the highest level by 1 h of treatment, and maintained high expression even after 24 h of cold treatment compared to the initial non-stress level (Fig 4A). Semi-quantitative RT-PCR results also showed the similar expression pattern (Fig 4B). However, *PpCBF3* expression did not respond to drought stress induced by PEG-6000 or salt stress in Kentucky bluegrass (S1 Fig).

### Growth and physiological characterization of freezing tolerance of *PpCBF3*-transgenic Arabidopsis

**Fig 5:**
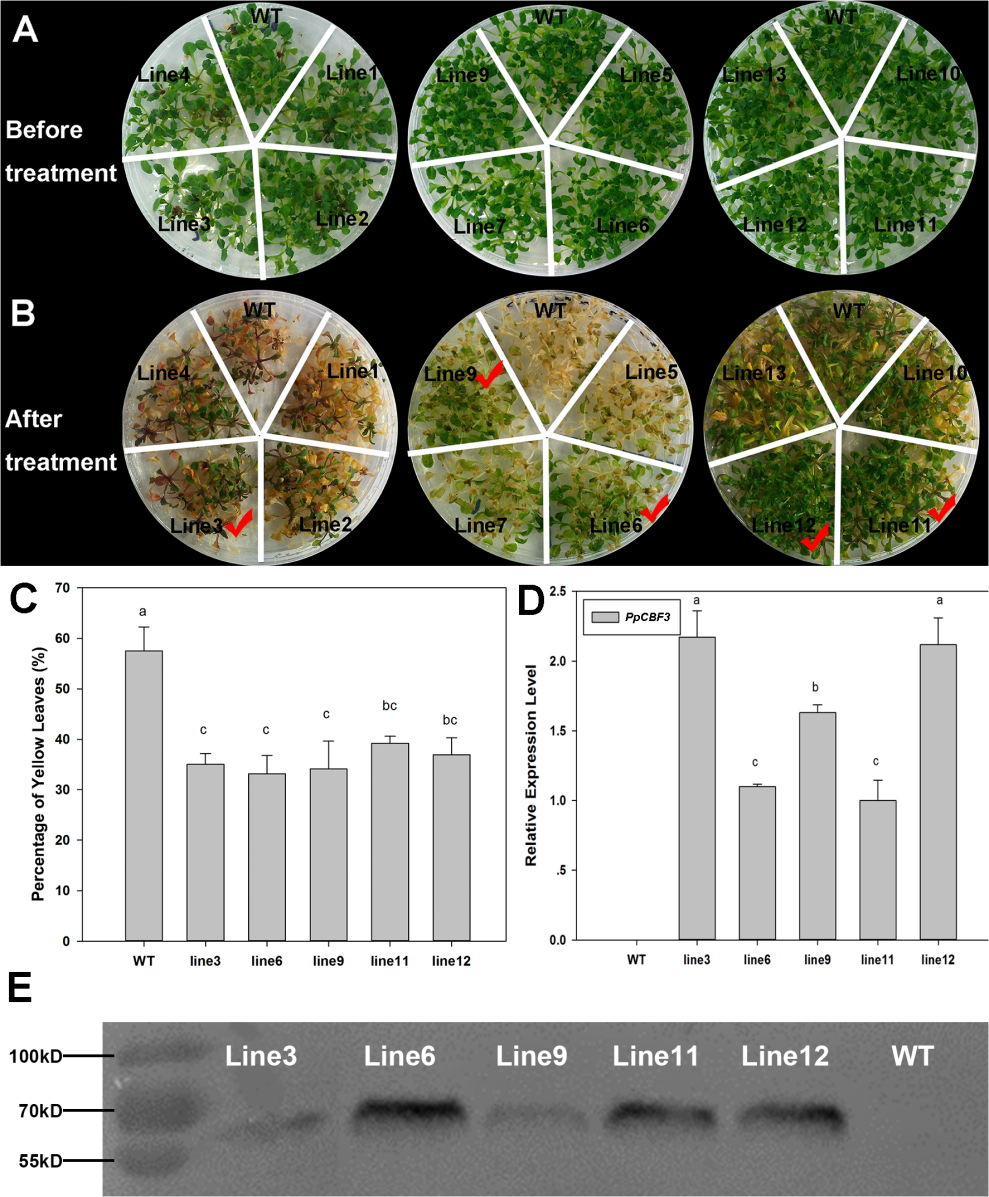
Expression analysis of PpCBF3 and phenotype of WT and transgenic lines under normal and cold treatment conditions. (A) Shows 4-week-old seedlings of WT and transgenic lines grow under normal condition. (B) Phenotype of WT and transgenic lines after -20°C freezing stress. Red tick represents the stable transformed lines finally selected for further analysis. (C) Percentage of chlorotic leaves is calculated in (B). More than half yellow color of one leaf is designated as chlorolic leaf. Values are means ± SD of fifteen independent plants. Different letters on the top of bars indicate significant differences (*P* < 0.05) between WT and transgenic lines under the same growth condition. (D) Detection of the relative mRNA expression level of *PpCBF3* in WT and transgenic lines grow under normal condition. (E) PpCBF3-GFP fused protein is detected by western blot in transgenic lines but not in WT.

To characterize the physiological functions of PpCBF3 involved in freezing tolerance, 16 transgenic lines of Arabidopsis were obtained (confirmed by PCR, S2 Fig) and 5 transgenic lines (Fig 5A–5C) exhibiting desirable phenotypes and improved freezing tolerance were selected for further experiments. qRT-PCR and Western blot analysis showed that *PpCBF3* was highly expressed in all 5 transgenic lines but not in WT (Fig 5D and 5E). The transgenic lines resumed growth following 7 d of recovery from 3 h exposure to -20°C (Fig 5A and 5B). The percentage of chlorotic leaves was significantly lower in the transgenic lines than in WT following freezing stress (Fig 5C).

**Fig 6:**
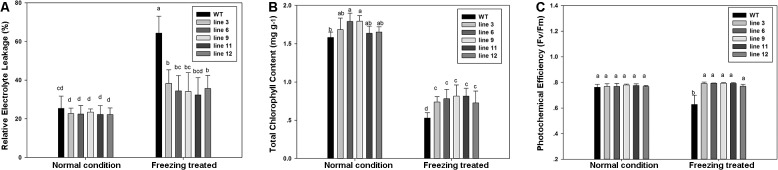
Physiological index including relative electrolyte leakage (EL), total chlorophyll content (Chl) and photochemical efficiency (Fv/Fm) in WT and transgenic plants under normal and freezing treated. Values are means ± SD of twelve independent plants. The same letters atop bars indicate that there is no significant difference at *P* < 0.05.

Under non-stress temperature, no differences in leaf EL were detected between the WT and transgenic lines (Fig 6A). Following freezing treatment, EL was significantly lower (about 35%) in all transgenic lines than in the WT (> 60%) (Fig 6A). Leaf chlorophyll content and Fv/Fm ratio decreased after freezing treatments in the WT and transgenic lines, but the transgenic lines maintained significantly higher chlorophyll content (approximately 0.8 in transgenic lines versus 0.5 in WT) and Fv/Fm (approximately 0.8 in transgenic lines versus 0.6 in WT) than WT (Fig 6B and 6C).

**Fig 7:**
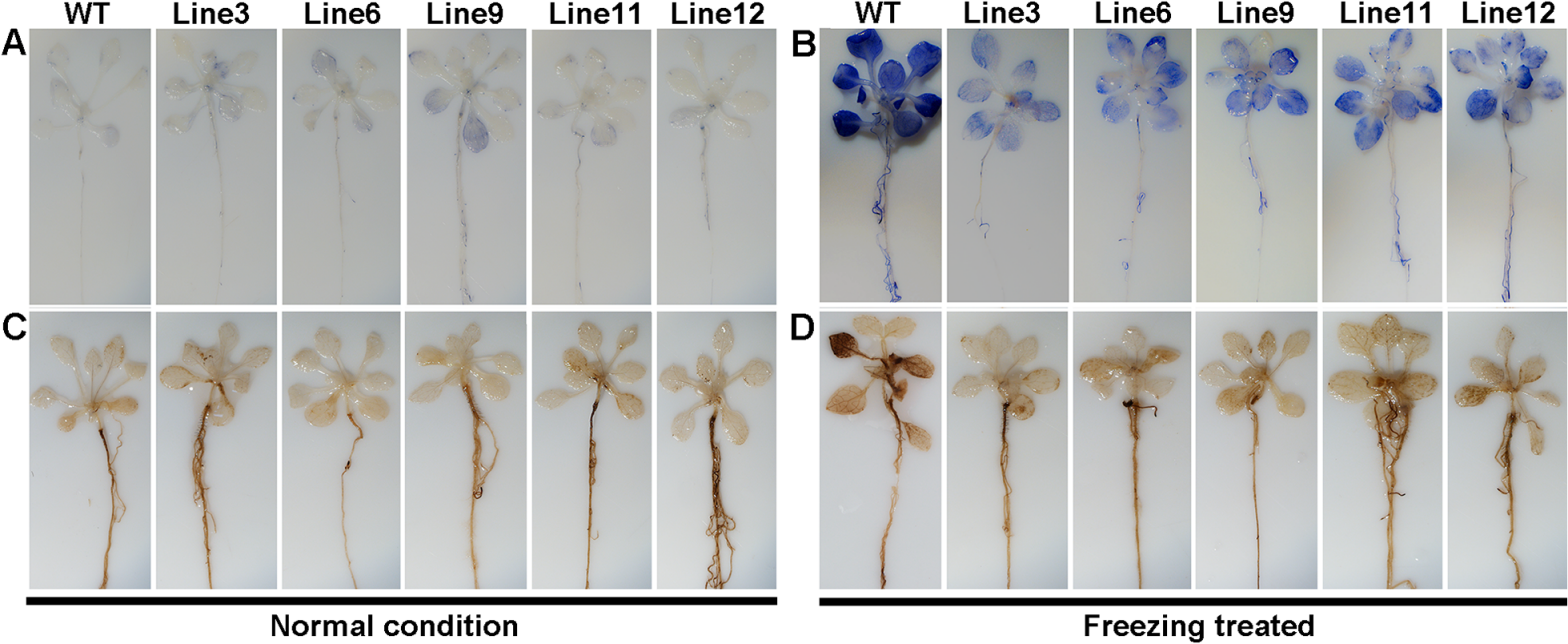
Detection of ROS in WT and transgenic Arabidopsis plants. (A) NBT staining for superoxide in WT and transgenic Arabidopsis under normal condition and (B) freezing stress. (C) DAB staining for hydrogen peroxide in WT and transgenic Arabidopsis under normal condition and (D) freezing stress.

To identify if transgenic plants accumulated less H_2_O_2_ and O_2_^.-^ under freezing stress at -20°C, leaves were stained with DAB and NBT. Leaves of the WT showed a dark blue color while those of all transgenic plants were stained light blue (Fig 7A and 7B), which indicated less accumulation of O_2_^.-^ in transgenic plants compare to the WT. Similarly, less H_2_O_2_ production was found in transgenic plants than in the WT, as shown by lesser intensity of brown staining of transgenic leaves (Fig 7C and 7D).

### Phenotypic alterations due to overexpression of *PpCBF3*

**Fig 8:**
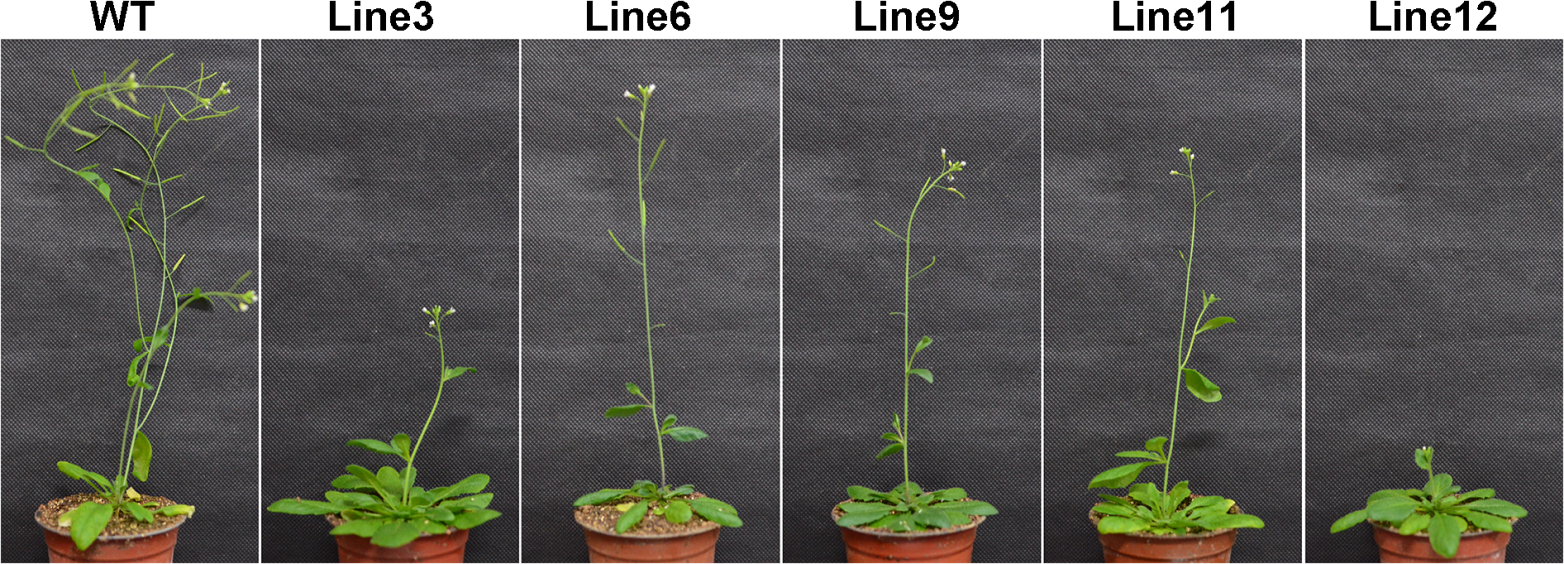
Relative mRNA expression level of downstream genes of DREB/CBF in WT and transgenic plants under normal and cold treatment (4°C). (A)-(D) Relative mRNA expression level of cold responsive gene (COR) in WT and transgenic plants under normal and cold treatment (4°C). (E) Relative mRNA expression level of P5CS controlling the rate- limiting step of glutamate- derived proline biosynthesis in WT and transgenic plants under normal and cold treatment (4°C).

In addition to differential phenotypic and physiological changes in response to freezing stress between the WT and transgenic plants, *PpCBF3*-transgenic plants grew slower than the WT under normal temperatures (Fig 8). The WT started to flower following 29 d of seedling emergence whereas transgenic lines did not flower until 34–43 d following seedling emergence (Table 1). The transgenic plants had an average 21 rosette leaves per plant before flowering compared with 13 rosette leaves in the WT (Table 1). After 6 weeks growth under non-stress temperature conditions, the WT bore many siliques and began to senescence. However, the transgenic plants were just beginning to flower or bore few siliques (Fig 8).

**Table 1:** Effects of PpCBF3 expression on flowering time and rosette leaf number.

Plants	Time to flowering (days)*	Rosette leaves per plant (n)^#^
WT	29	13.0±1.93 (14)^b^
Line 3	43	21.8±1.98 (13)^a^
Line 6	34	20.7±2.26 (10)^a^
Line 9	36	21.5±3.14 (11)^a^
Line 11	37	21.6±1.67 (10)^a^
Line 12	43	21.5±1.97 (10)^a^

^a, b^Means followed by the same letter are not significantly different at α = 0.05 based on single degree of freedom contrasts from within an analysis of variance.

^*^Period of time between plants germination and appearance of first flower buds in a population of “n” plants.

^#^Means representing the rosette leaves number of WT and 5 transgenic lines divided by the number “n”.

### Expression of DREB/CBF down-stream target genes induced by *PpCBF3*

**Fig 9:**
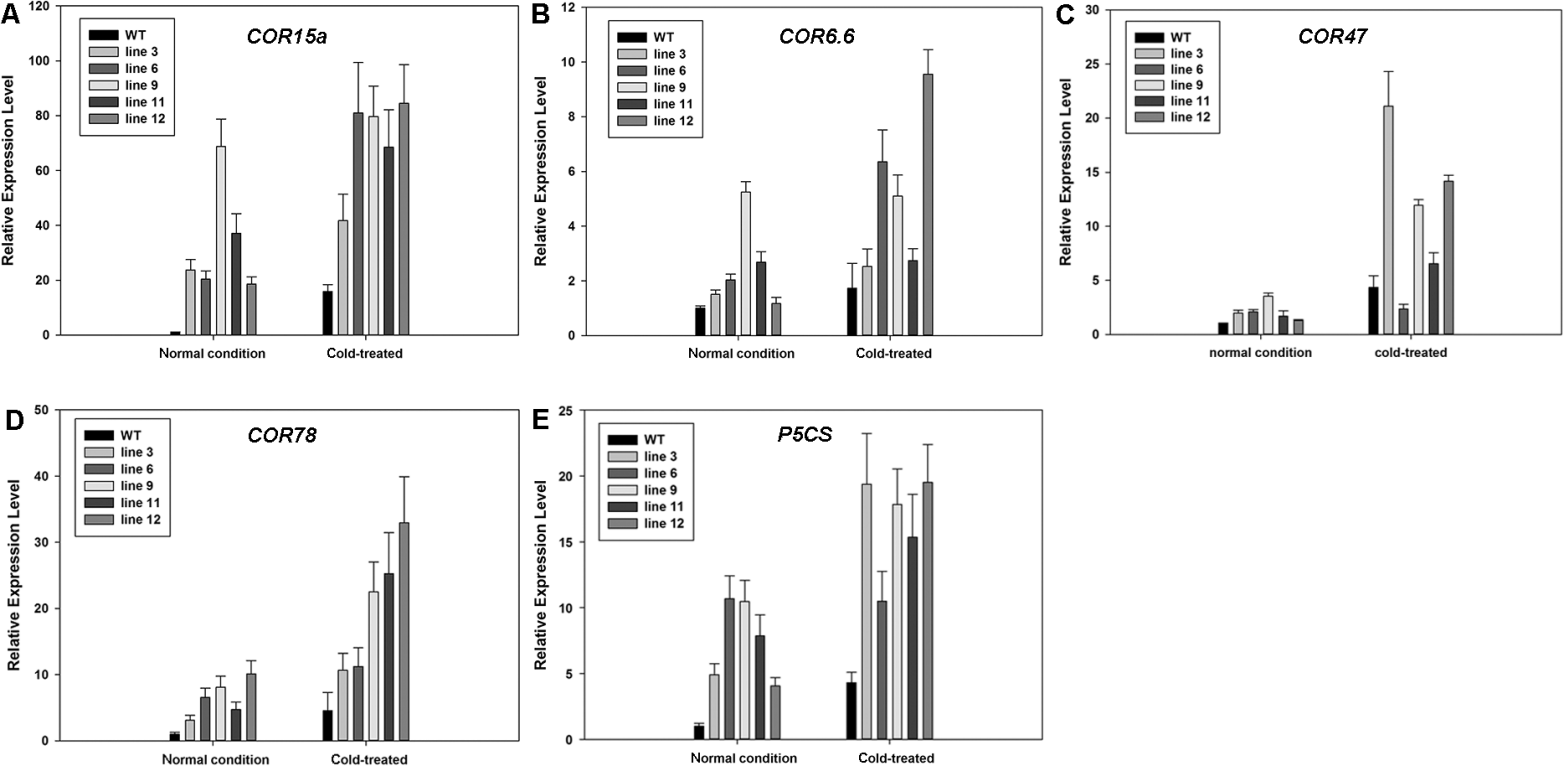
Growth characteristic of 6-week-old WT and transgenic Arabidopsis under normal growth condition. All transgenic plants show late flowering phenotype compared with WT. The growth chamber condition is set at 23 ^**o**^C, 16h/8h light/dark, 70% humidity, 120 μmol m^**-2**^ s^**-1**^ light density.

To determine whether overexpressing *PpCBF3* may activate DREB1/CBF downstream target genes such as *COR15a*, *COR6*.*6*, *COR47* and *COR78*, the relative expression level of target genes was determined by qRT-PCR analysis in transgenic plants and WT under normal temperature and 4°C treatments. The results showed that under normal temperature, these genes were expressed in transgenic plants but exhibited lower level in WT (Fig 9A–9D). After 4°C treatment, they were up-regulated in both the WT and transgenic plants but these target genes showed higher expression levels in transgenic plants compared to WT. In addition, relative expression level of Δ^1^-pyrroline-5-carboxylate synthase (*P5CS*) was detected which also showed higher transcript levels in transgenic plants than the WT both under normal and cold stress conditions (Fig 9E).

## Discussion

### PpCBF3 belongs to DREB/CBF family and specifically responsive to cold stress

The *PpCBF3* isolated from Kentucky bluegrass contains a conserved AP2 DNA binding domain and belongs to the DREB1/CBFs subfamily of AP2 superfamily. Previous study showed that the DREB subfamily was further divided into six small groups from A-1 to A-6 [37]. The presence of two signature sequences, one was ‘DSAW’ motif at the end of AP2 domain and the other was ‘LWSY’ motif at the end of C-terminal region, testified that PpCBF3 belonged to the A-1 group. Phylogenetic analysis showed that PpCBF3 was clustered with LpCBF3 and OsDREB1A. PpCBF3 contains a putative nuclear localization signal (NLS) upstream of the AP2 domain and was localized in the cell nucleus. This was consistent with the transcription activator function of CBF genes as found by others [38]. These results suggested that PpCBF3 is a transcription factor.

The CBF genes are known to be responsive to various abiotic stresses [5,39,40]. In this study, *PpCBF3* expression responded rapidly to chilling stress (4°C) but did not change in response to drought stress induced by PEG-6000 or salt stress (S1 Fig). It has been reported that not all of the six CBF homologs in Arabidopsis are functionally related to cold tolerance. For example, CBF4 is mainly participated in drought resistance whereas the other two homologs, DDF1 and DDF2, take part in the regulation of gibberellins biosynthesis and salinity tolerance [22,41]. Recently, Kidokoro et al. (2015) [8] identified 14 DREB1-type transcription factors, GmDREB1s, from the soybean genome database and found DREB1 proteins play important roles in heat stress responses. The current results suggested that the *PpCBF3* in the DREB1/CBF A-1 subfamily isolated from Kentucky bluegrass was specific to cold stress responses. Other members of DREB family in Kentucky bluegrass could play roles in responses to other abiotic stresses, such as drought or salinity stress, and deserves further investigation.

### Enhanced tolerance to severe freezing stress of *PpCBF3-transgenic* Arabidopsis associated with up-regulation of downstream cold-related genes

Overexpression of DREB1/CBF transcription factors in regulating cold tolerance has been reported in agronomic crops and the model plant Arabidopsis [13–16,42]. In Arabidopsis, the expression of three DREB1 genes (*CBF1*, *CBF2* and *CBF3*) was strongly induced by cold stress [43]. These DREB1 genes predominantly acted in cold stress-inducible gene expression because of the enrichment of CRT/DRE in the promoters of cold stress-responsive genes in Arabidopsis [44]. In our study, transgenic Arabidopsis plants overexpressing *PpCBF3* exposed to -20°C showed significant improvement in freezing tolerance compared to the WT as manifested by increased chlorophyll content and Fv/Fm, as well as the decreases in the percentage of chlorotic leaves and EL in transgenic plants. The growth and physiological analyses strongly suggested that over-expressing *PpCBF3* played positive roles in alleviating damages from extremely low freezing temperature (-20°C), unlike previous studies which have mostly reported improved tolerance to chilling stress or mild freezing stress [13,15,18,19,25].

Cellular injuries of cold stress are related to ROS (O_2_^.-^ and H_2_O_2_) production that causes oxidative damage [45]. The improvement of freezing tolerance by *PpCBF3* overexpression was found to be related to the suppression of O_2_^.-^ and H_2_O_2_ accumulation as shown by the NBT and DAB staining, respectively. In addition, it is reported that high concentrations of proline is involved in stress tolerance [46–48]. Indeed, expression level of *P5CS* which encodes an enzyme involved in proline synthesis was up-regulated in transgenic Arabidopsis plants, although proline content was not determined in this study. Our results strongly demonstrated that PpCBF3 could play important roles in protecting leaf damages from freezing stress by protecting photosynthetic components (chlorophyll content and photochemical reactions), cell membranes, and suppressing ROS formation, as well as up-regulation of proline synthesis, ultimately leading to improved plant growth and survival under extremely low freezing temperature.

Through the binding of DRE/CRT promoters, CBFs activate many downstream COR genes encoding hydrophilic polypeptides which enhance tolerance to low temperature in plants [19]. Consistently, the expression of COR genes including *COR15a*, *COR6*.*6*, *COR47* and *COR78* were constitutively activated by *PpCBF3* in transgenic plants and up-regulated under cold stress. It is notable that the fold change of *COR15a* expression in transgenic plants was greater than that of other COR genes under normal and freezing stress, suggesting that *COR15a* could play a prominent role in freezing tolerance of transgenic plants overexpressing *PpCBF3*. It has been reported that in transgenic Arabidopsis plants, overexpressing *COR15a* improved freezing tolerance of chloroplasts frozen in situ (-4°C and -5°C) and of protoplasts frozen in vitro (between -5°C and -8°C) [49]. However, *COR15a* expression in transgenic Arabidopsis has no significant effect on the survival of whole frozen plants. Several *COR* genes (i.e. COR6.6a and COR47) are cooperatively stimulated along with *COR15a* when subjected to low temperature [50,51], which suggests that these *COR* genes might act in concert with each other to increase tolerance to freezing stress in plants. It is postulated that expression of the entire batch of *COR* genes activated by *PpCBF3* would have greater effects on freezing tolerance than *COR15a* expression alone.

### Phenotypic alterations due to overexpression of *PpCBF3* in Arabidopsis

Previous studies have reported constitutive over-expression of DREB1/CBF genes resulted in stunted shoot growth, delayed flowering, and lower yields, all of which are considered negative characteristics for agronomic crop species [3,13–15,52]. Many attempts have been reported to minimize the negative effects of DREB1/CBF over-expression by using stress-inducible promoters [14,15,20,42,53]. Overexpression of *PpCBF3* in Arabidopsis also slowed growth and delayed flowering in transgenic plants. Unlike agronomic crops, slower vertical growth and delayed flowering are desirable traits in perennial grasses used as turfgrass. Slow-growing turfgrasses require less-frequent mowing, irrigation, and fertility inputs while the presence of flowering reduces turf visual quality and uniformity [26]. Therefore, *PpCBF3* could be potentially useful for genetic engineering of turfgrass improvement to freezing tolerance and developing other desirable traits, such as slowed growth and late flowering.

In summary, *PpCBF3* isolated from cold-tolerant Kentucky bluegrass was characterized as a DREB1/CBF transcriptional factor and was responsive specifically to cold stress. The physiological and gene expression analysis of transgenic Arabidopsis overexpressing *PpCBF3* confirmed *PpCBF3* serves roles in regulating plant tolerance to severe freezing stress and was associated with the up-regulation of downstream genes (*COR15a*, *COR6*.*6*, *COR47*, *COR78* and *P5CS*) involved in cold tolerance. The positive physiological effects of *PpCBF3* for freeing tolerance and the induction of desirable turfgrass phenotypic traits suggested that *PpCBF3* could be a useful candidate gene for genetic modification of perennial grasses for high turf quality and improved freezing tolerance.

## Supporting Information

S1 Fig Relative mRNA expression level of *PpCBF3* in leaves of *Poa pratensis* under 20% PEG and 150 mM NaCl treatment. Values are means ± SD of three independent experiments. The same letters atop bars indicate that there is no significant difference at *P* < 0.05.
(TIF)

S2 Fig PCR detection of positive T_1_ transgenic plants. WT plants are used as negative control. Numbers indicate transgenic line. Only 13 lines are showned here.
(TIF)

S1 Table Sequences of primers used in this article. (XLSX)
