# Supplementary figures and images for: PpCBF3 from Cold-Tolerant Kentucky Bluegrass Involved in Freezing Tolerance Associated with Up-Regulation of Cold-Related Genes in Transgenic Arabidopsis thaliana
Source: PLoS One. 2015 Jul 15;10(7):e0132928. doi: 10.1371/journal.pone.0132928 (PMC4503346; doi:10.1371/journal.pone.0132928)

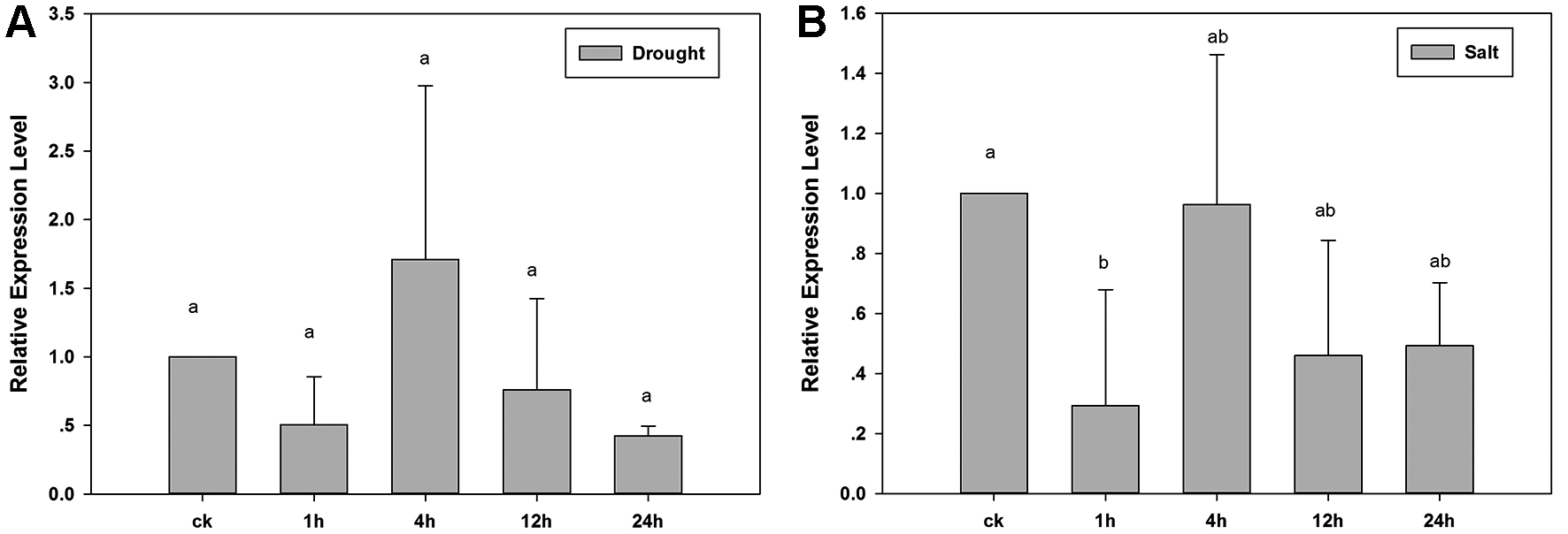

Supplement: S1 Fig — Values are means ± SD of three independent experiments. The same letters atop bars indicate that there is no significant difference at P < 0.05. (TIF) [file pone.0132928.s001.tif]

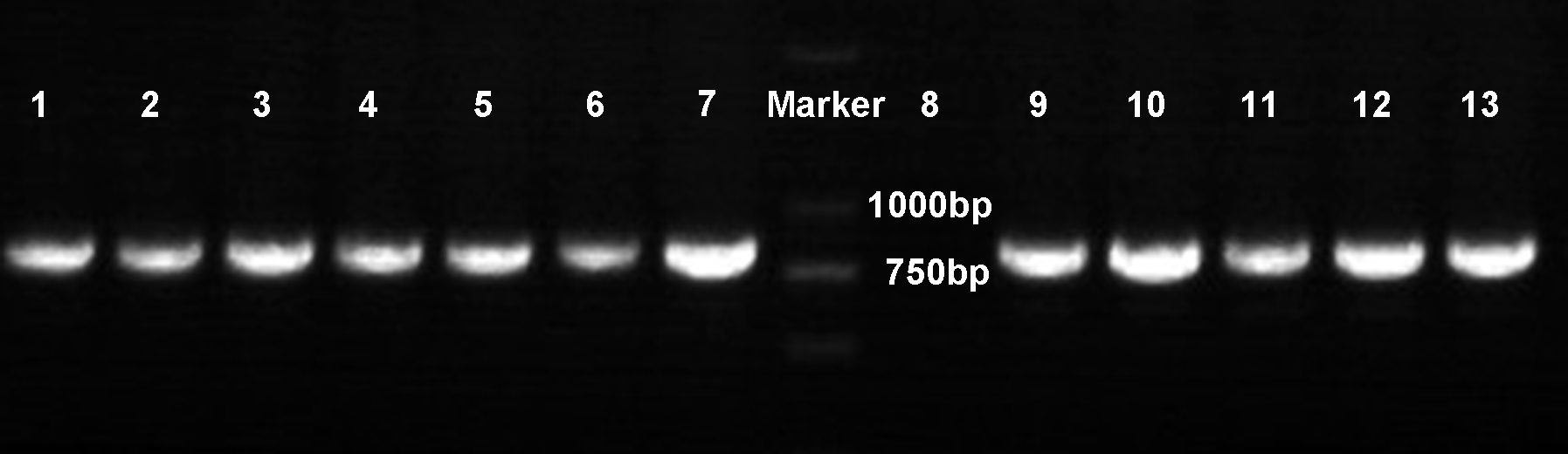

Supplement: S2 Fig — WT plants are used as negative control. Numbers indicate transgenic line. Only 13 lines are showned here. (TIF) [file pone.0132928.s002.tif]
